# Extra-Uterine Pregnancy

**Published:** 1876-04

**Authors:** 


EXTRA-UTERINE PREGNANCY—Its Causes, Species, Pathological Anatomy, Clinical History, Diagnosis, Prognosis, and Treatment. By John S. Parry, M.D., Obstetrician to Philadelphia Hospital, Physician for Diseases of Women in Presbyterian Hospital, Fellow of the College of Physicians of Philadelphia, etc. Philadelphia: Book, cloth, pp. 276. Henry C. Lea.

It is impossible, with our space, to make a complete analytical notice of this volume. This work is the result of a careful analysis of several hundred cases of extra-uterine pregnancy, with practical deductions therefrom. We can only cheerfully commend it to our readers who are unacquainted with its merits, as a useful light in this department of science.

